# L-Carnitine augments probenecid anti-inflammatory effect in monoiodoacetate-induced knee osteoarthritis in rats: involvement of miRNA-373/P2X7/NLRP3/NF-κB milieu

**DOI:** 10.1007/s10787-023-01376-w

**Published:** 2023-11-23

**Authors:** Rawan Mahfouz, Safaa H. El-Rewini, Asser I. Ghoneim, Eman Sheta, Mennatallah A. Ali, Sherihan Salaheldin Abdelhamid Ibrahim

**Affiliations:** 1 Department of Pharmacology and Therapeutics, Faculty of Pharmacy, Pharos University in Alexandria (PUA), Canal El- Mahmoudia Street, Smouha, Alexandria Egypt; 2 Department of Pharmacology, Faculty of Medicine, Alexandria University, Alexandria, Egypt; 3 Department of Pharmacology and Toxicology, Faculty of Pharmacy, Damanhour University, Damanhour, Egypt; 4 Department of Pathology, Faculty of Medicine, Alexandria University, Alexandria, Egypt

**Keywords:** L-Carnitine, Probenecid, Monoiodoacetate-induced knee osteoarthritis, miRNA-373, P2X7/NLRP3/NF-κB pathway

## Abstract

Osteoarthritis (OA) is a degenerative joint disease, whereas the underlying molecular trails involved in its pathogenesis are not fully elucidated. Hence, the current study aimed to investigate the role of miRNA-373/P2X7/NLRP3/NF-κB trajectory in its pathogenesis as well as the possible anti-inflammatory effects of probenecid and l-carnitine in ameliorating osteoarthritis via modulating this pathway. In the current study, male Sprague Dawley rats were used and monoiodoacetate (MIA)-induced knee osteoarthritis model was adopted. Probenecid and/or L-carnitine treatments for 14 days succeeded in reducing OA knee size and reestablishing motor coordination and joint mobility assessed by rotarod testing. Moreover, different treatments suppressed the elevated serum levels of IL-1β, IL-18, IL-6, and TNF-α via tackling the miRNA-373/P2X7/NLRP3/NF-κB, witnessed as reductions in protein expressions of P2X7, NLRP3, cleaved caspase-1 and NF-κB. These were accompanied by increases in procaspase-1 and IκB protein expression and in miRNA-373 gene expression OA knee to various extents. In addition, different regimens reversed the abnormalities observed in the H and E as well as Safranin O-Fast green OA knees stained sections. Probenecid or l-carnitine solely showed comparable results on the aforementioned parameters, whereas the combination therapy had the most prominent effect on ameliorating the aforementioned parameters. In conclusion, l-carnitine augmented the probenecid’s anti-inflammatory effect to attenuate MIA-induced osteoarthritis in rats by provoking the miRNA-373 level and inhibiting the P2X7/NLRP3/NF-κB milieu, leading to the suppression of serum inflammatory cytokines: IL-1β, IL-18, IL-6, and TNF-α. These findings suggest the possibility of using probenecid and l-carnitine as a useful therapeutic option for treatment of osteoarthritis.

**Graphical abstract:**

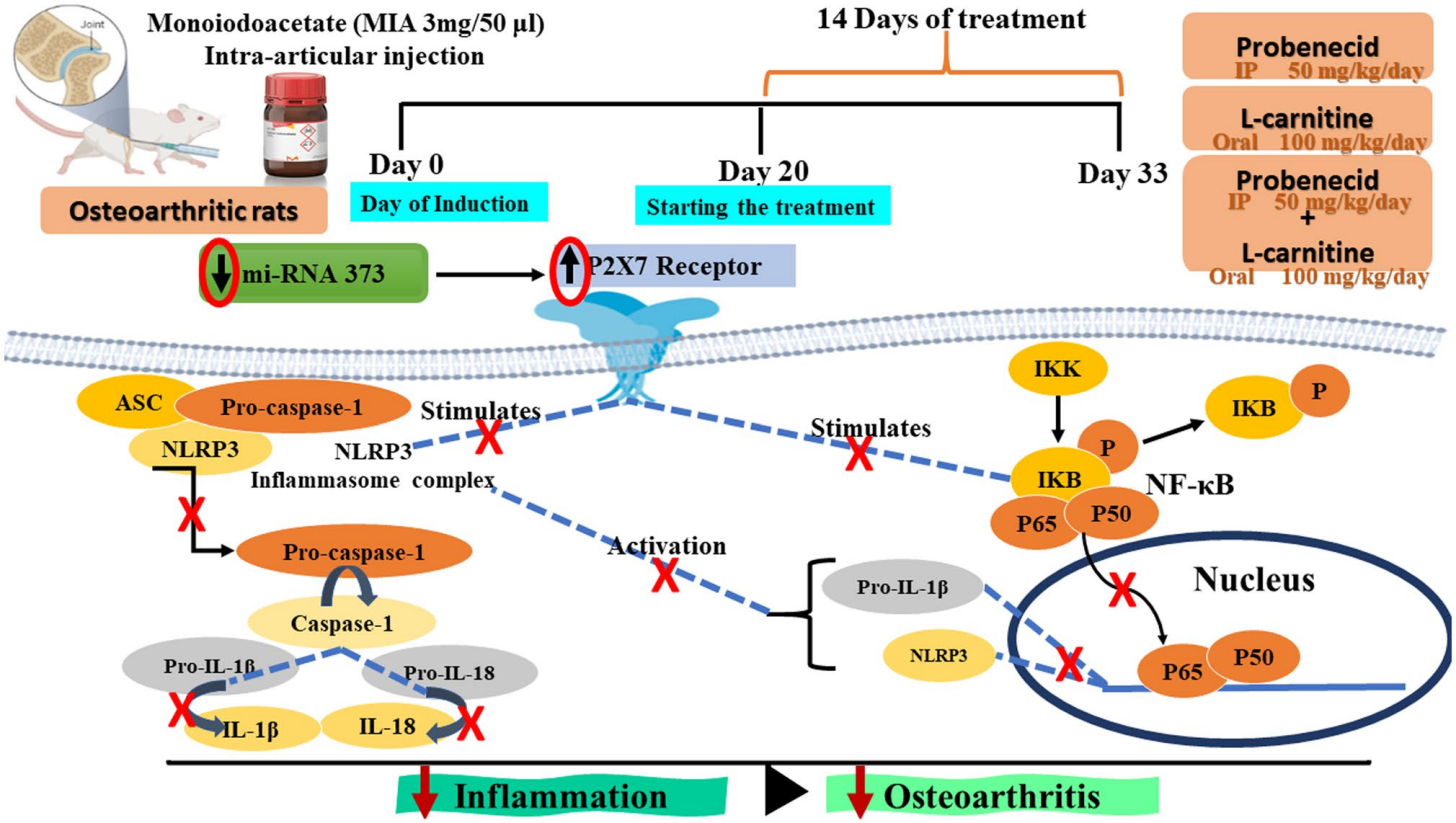

## Introduction

Osteoarthritis (OA) is a degenerative joint disease with an increasing prevalence due to many factors such as age, female gender, obesity, and injuries to the joint (Yao et al. 2023). The OA is characterized by a progressive inflammation, which is a complicated biological reaction of injured tissues, that leads to structural changes such as degeneration of articular cartilage, osteophyte formation, and subsequent joint space narrowing as well as involvement of subchondral bone of synovial joints leading to pain during weight-bearing activities (Sanchez-Lopez et al. 2022; Faour et al. 2016).

An injury caused by trauma, an infection, a post-ischemic condition, a toxin, or an autoimmune reaction can all trigger inflammation, a complicated biological reaction of the injured tissues.

Recently, it has been illustrated that inflammatory cytokines play a key role in the pathogenesis of the disease. Pro-inflammatory cytokines such as tumor necrosis factor (TNF)-α, interleukin-1β (IL-1β), IL-6, and IL-18 are produced by chondrocytes in the synovium, which in turn trigger the production of factors such as matrix metalloproteinases (MMPs) that further augment cartilage degradation and apoptosis of chondrocytes (Sanchez-Lopez et al. 2022; Chow and Chin 2020).

New approaches in OA management are striving to target the dysregulated intracellular pathways that are activated in OA cartilage tissues and have been attributed to pain and inflammation such as the purinergic 2 X7 receptor (P2X7R)/inflammasome (NLRP-3)/nuclear factor (NF)-κB pathway (Hu et al. 2016).

The P2X7 purinergic receptors belong to one of the P2X ion channel receptor family members that have been suggested to mediate pain and inflammation in OA (Chow and Chin 2020). In addition, other studies illustrated that activation of P2X7 receptors by elevated levels of ATP mediated by pannexin channels can promote the activation of NF-κB and subsequent release of IL-1β, IL-6, and TNF-α (Zeng et al. 2019; El-Tedawy et al. 2020).

In addition, P2X7 activation can decrease the intracellular K^+^ concentration, which permits the nucleotide-binding and oligomerization domain-like receptor containing protein 3 (NLRP3) inflammasome assembly and release of IL‐1β and IL‐18 (Zeng et al. 2019). Therefore, tackling the P2X7 receptor signal pathway might postulate a promising treatment strategy for OA.

The microRNAs (MiRNAs), which act as fine-tuning regulators of gene expression and constitute an additional layer of regulation at the post-transcriptional level, are shown to be extensively implicated in OA disease. It was previously reported that downregulated expression of (miR-373) in the chondrocytes from OA patients causes chondrocyte proliferation and an increase in inflammatory factors such as IL-6 via increasing the expression level of P2X7R (Zhang et al. 2018).

Probenecid is an inhibitor of organic anion transporters in renal proximal tubes to prevent reuptake of uric acid from urine and is predominately used as a second-line treatment of gout in humans (Robbins et al. 2012). A previous study showed that probenecid was effective at dampening hyperinflammation and severe influenza disease in mice by targeting the P2X7 receptor (Rosli et al. 2019). In addition, it was suggested that it could impair the ATP release channel pannexin-1 (Silverman et al. 2008), which can directly interact with human and rodent P2X7 (Pelegrin and Surprenant 2006; Silverman et al. 2009).

L-Carnitine [LC], the bioactive form of carnitine, is an endogenous branched nonessential amino acid derivative that plays a critical role in energy production by transporting long-chain fatty acids from the cytoplasm to mitochondria so they can be oxidized to produce energy. L-Carnitine has been shown to offer a great therapeutic potential against several chronic conditions including cardiovascular, diabetes, neurodegenerative, and inflammatory diseases. High doses can cause GIT upset, a “fishy” body odor, and rarely muscle weakness and seizures (D’Antona et al. 2014). Moreover, its anti-inflammatory and antioxidant effects have been illustrated in many studies (Ali et al. 2016; Keshani et al. 2022; Fathizadeh et al. 2020).

Hence, the current study aimed to investigate the anti-inflammatory effects of each of probenecid and l-carnitine alone and in combination in ameliorating OA via modulating miR-373/P2X7/NLRP3/NF-κB trajectory.

## Materials and methods

### Drugs and chemicals

Monoiodoacetate (Cat# 305533, Sigma-Aldrich, USA) was used for the induction of knee OA in rats. The drugs used in the study were Probenicid (Cat# P8761, Sigma-Aldrich, USA) and L-carnitine (Cat# C0158, Sigma-Aldrich, USA). Each assay’s kits and antibodies are mentioned below along with details.

### Animals

Adult male Sprague Dawley albino rats weighing 150–175 gm were procured from the animal house of Pharos University in Alexandria. The rodents were given unrestricted access to food pellets and water and kept for one week to have an acclimation period. All experimental procedures were carried out in accordance with the National Institutes of Health (NIH) guidelines for the Care and Use of Laboratory Animals, the Animal Research: Reporting of In Vivo Experiments (ARRIVE) guidelines, and were approved by the "Unit of Research Ethics Approval Committee, Pharos University in Alexandria" (PUA-01202104061021).

### Monoiodoacetate-induced knee osteoarthritis model

The monoiodoacetate (MIA) was dissolved in 0.9% sterile phosphate-buffered saline (PBS). Then, induction of unilateral knee OA was done by giving an intra-articular injection of MIA (3 mg/50 μl). In the control group, 50 μl of sterile PBS was injected. The animals were given a week to acquire OA symptoms. After 20 days, an osteoarthritic model emerged (Hanafy and El-Ganainy 2020).

### Experimental groups

**Fig. 1:**
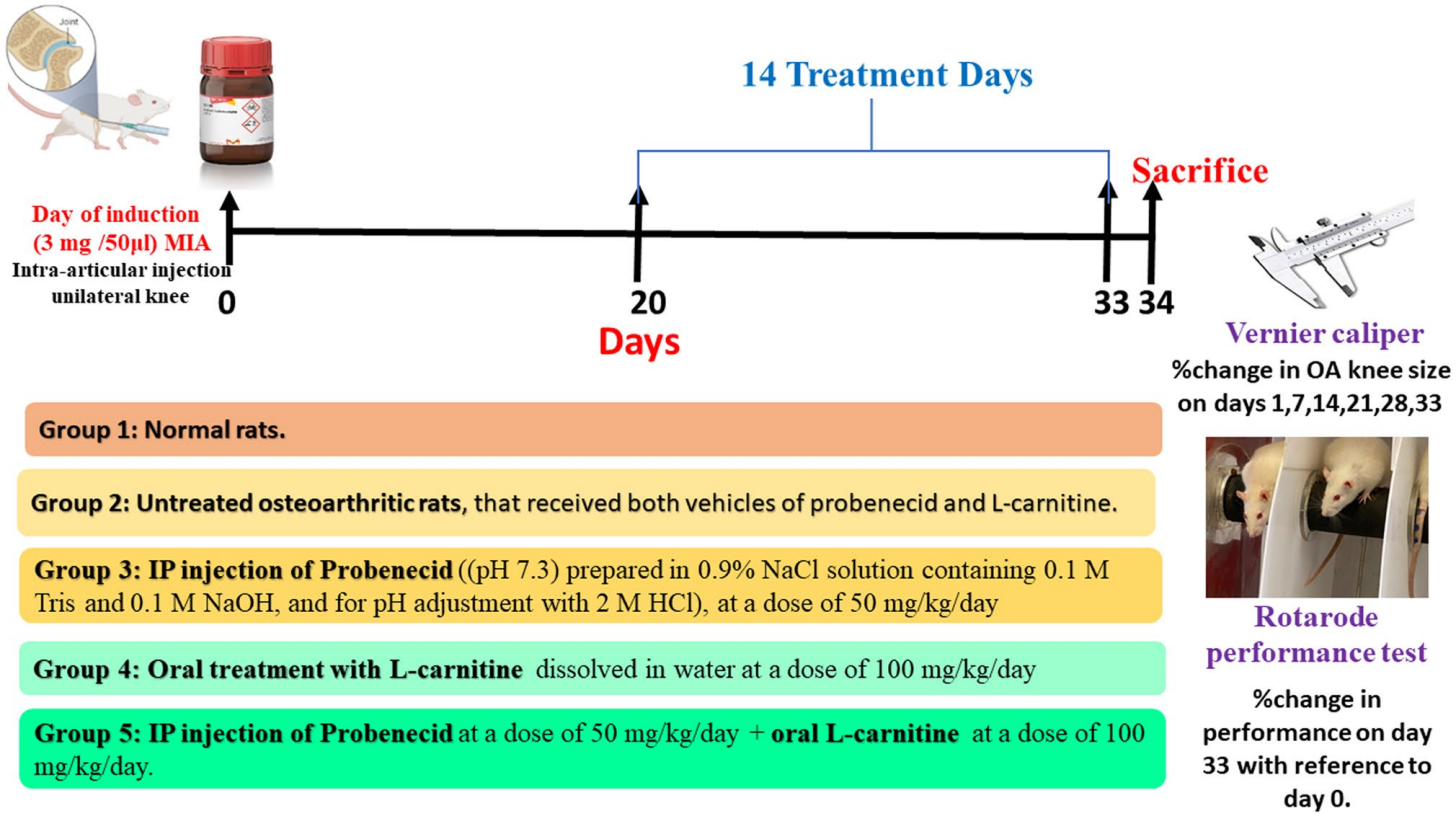
Timeline for drug administration in the experimental design

The animals were randomly and equally divided into 5 groups (each group = 6 rats). Group 1 were healthy rats. Group 2 consisted of untreated osteoarthritic rats that received both vehicles of probenecid and L-carnitine. Group 3 consisted of osteoarthritic rats that were treated via intraperitoneal injection of probenecid ((pH 7.3) prepared in 0.9% NaCl solution containing 0.1 M Tris and 0.1 M NaOH, and for pH adjustment with 2 M HCl), at a dose of 50 mg/kg/day (Sigma-Aldrich, St. Louis, MO, USA) (Zhang et al. 2019). Group 4 consisted of osteoarthritic rats that were treated orally with L-carnitine dissolved in water at a dose of 100 mg/kg/day (Sigma-Aldrich, St. Louis, MO, USA) (20). Group 5 consisted of osteoarthritic rats that were treated with both drugs at the aforementioned doses. Tested drugs were administered daily for 14 days from the 20th day till the end of the study Fig. 1.

### Assessment of osteoarthritis progression

#### Evaluation of OA knee size

The diameters of all rats’ right (OA) and left (normal) knees were measured on day 0 before the induction, as well as on days 1, 7, 14, 21, 28, and 33 after the induction with MIA. Knee diameters were measured using a Vernier caliper, and percentage change was calculated (Ali et al. 2016; Domiati et al. 2016).

$$ {\text{Percentage change in OA knee size }} = \, ({\text{OA knee size - normal knee size}})/{\text{normal knee size }} \times \, 100 $$

#### Behavioral test

The time to fall was calculated using an accelerated rotarod performance test. The rats were subjected to a one-minute acceleration of 5 to 16 rpm, while the time of initial failure to stay atop the rod was recorded and the average of three different readings was determined. Rotarod testing was performed on days 0 and 33. The percentage change in performance was determined with reference to day zero numbers (Hanafy and El-Ganainy 2020).

### Serum inflammatory cytokines

Animals were euthanized with thiopental sodium (50 mg/kg). Blood samples were collected from the posterior vena cava, serum was separated and used for the determination of TNF‐α (Cat# ab236712, Abcam, USA), IL‐1β (Cat# BMS630, Invitrogen, USA), IL‐6 (Cat# BMS625, Invitrogen, USA) and IL-18 (Cat# KRC2341, Invitrogen, USA). All the procedures of the assays were done according to the ELISA kit manufacturers’ instructions.

### Western blot analysis

This approach was utilized to determine the protein expression of P2X7R, NLPR3, Pro-Caspase-1, Cleaved caspase-1, p-NF-κB p65, and IκB in cartilage tissues of the knee joint of osteoarthritic-treated and untreated rats. Knee Cartilages were excised, homogenized, and macerated in lysis buffer. Then, supernatant was obtained by centrifugation at 12,000 xg for 5 min at 4 °C. The BCA assay kit (Bio-Rad Labs assay kit, USA) was used to determine the total amount of proteins. Furthermore, 30 µg of protein was incubated for 20 h with the primary antibodies mentioned in Table 1. This was followed by incubation with goat anti-rabbit-horseradish peroxidase (HRP) secondary antibody. A Chemi-Doc imager was used to acquire the band intensities after applying the Western Lightning Plus ECL Chemiluminescence substrates (Perkin Elmer, USA).

**Table 1:** Primary and Secondary antibodies used in the western bolt analysis

*Primary antibodies against*	
P2X7R	Cat no. # PA5-29,274, Invitrogen, Thermofisher Scientific, USA
NLPR3	Cat no. # ab263899, Abcam, USA
Pro-Caspase-1	Cat no. # 2225, Cell Signaling Technology, USA
Cleaved caspase-1	Cat no. #67,314, Cell Signaling Technology, USA
p-NF-κB p65	Cat no. # ab76302, Abcam, USA
IκBα	Cat no. # 4812; Cell Signaling Technology, USA
β-actin	Cat no. # sc-47778, Santa Cruz, USA
*Secondary antibodies against*	
Horse radish peroxidase (HRP)	Cat no. # 31,460, Thermofisher Scientific, USA

### Quantitative reverse transcription polymerase chain reaction (RT-PCR)

We used a TRIzol and RNA purification kit (Cat no. # 12,183,555, Invitrogen, Thermofisher Scientific, USA) to extract total RNA from knee cartilages. Reverse transcription was performed using Prime Script™ RT Master Mix (Cat no. # RR036B, Takara, USA). This was followed using TB Green Premix Ex Taq II (Cat no. # RR820A, Takara, USA) with forward and reverse primers mentioned in Table 2 for performing real-time PCR. The 2^− ΔΔCt^ method was used to determine miR-373 transcription levels using U6 as the reference gene (Bai et al. 2018).

**Table 2:** Forward and reverse primers of miR-373 and U6

Gene	Forward sequence	Reverse sequence
miR-373	5′-ATTTTGGTTAATACGGTGAAATTTC-3′	5′-CTATCGCCCAAACTAAAATACGAT-3′
U6	5′-CTCGCTTCGGCAGCACA-3′	5′-ACGCTTCACGAATTTGCGT-3′

### Histopathological examinations

The right knee joint (lower end femur and upper end of tibia) from each rat was excised. It was fixed in formalin for 24 h, followed by decalcification. Joints were then cut longitudinally and placed in cassettes. They were processed into paraffin blocks. Using a Leica RM2235 rotatory microtome, two five-micron thick sections were cut and mounted on glass slides. One section was stained by hematoxylin and eosin (H&E) stain to identify different anatomical sites of the joints. Synovitis was evaluated by the synovial membrane scoring system which includes evaluation of synovial membrane hyperplasia, lymphocytic/plasmacytic inflammation, sub-synovial fibrosis, and vascularity. Each parameter was individually assessed and graded (0–3) then a total score out of 12 was calculated (Mainil-Varlet et al. 2013).

The other five microns section was stained by safranin O-fast green stain for cartilage (SOC-IFU, ScyTek Laboratories, Inc., USA) according to the manufacturer’s manual. The stained section was used to grade the cartilage injury and proteoglycan content according to the modified Mankin scoring system. The scoring system includes 4 different parameters (cartilage structure, chondrocytes architecture, matrix staining, and tidemark integrity) with a total score out of 13 (Mankin et al. 1971). The photographs were obtained with a Leica EC4 digital microscope camera from Germany, at the image analysis unit of the pathology department, faculty of Medicine, Alexandria University.

### Statistical analysis

To analyze the acquired data, we utilized one-way ANOVA followed by a Tukey post hoc test for multiple comparisons. Analysis of % change in OA knee edema size in comparison to the other normal knee on days 0,1,7,14,21,28,33 was carried out using two-way ANOVA followed by Bonferroni’s test. The data were presented as the mean ± standard deviation (SD) of six observations. The synovitis and modified Mankin scores were analyzed using the Kruskal–Wallis test followed by Dunn’s post-test (*n* = 6). Statistical correlations between various factors were calculated using the Pearson coefficient test. A P value of less than 0.05 was regarded as significant. All analysis was done using GraphPad Prism 0.8 (GraphPad Software Inc., USA).

## Results

### Probenecid and/or l-carnitine mitigated the MIA-provoked OA knee size

**Fig. 2:**
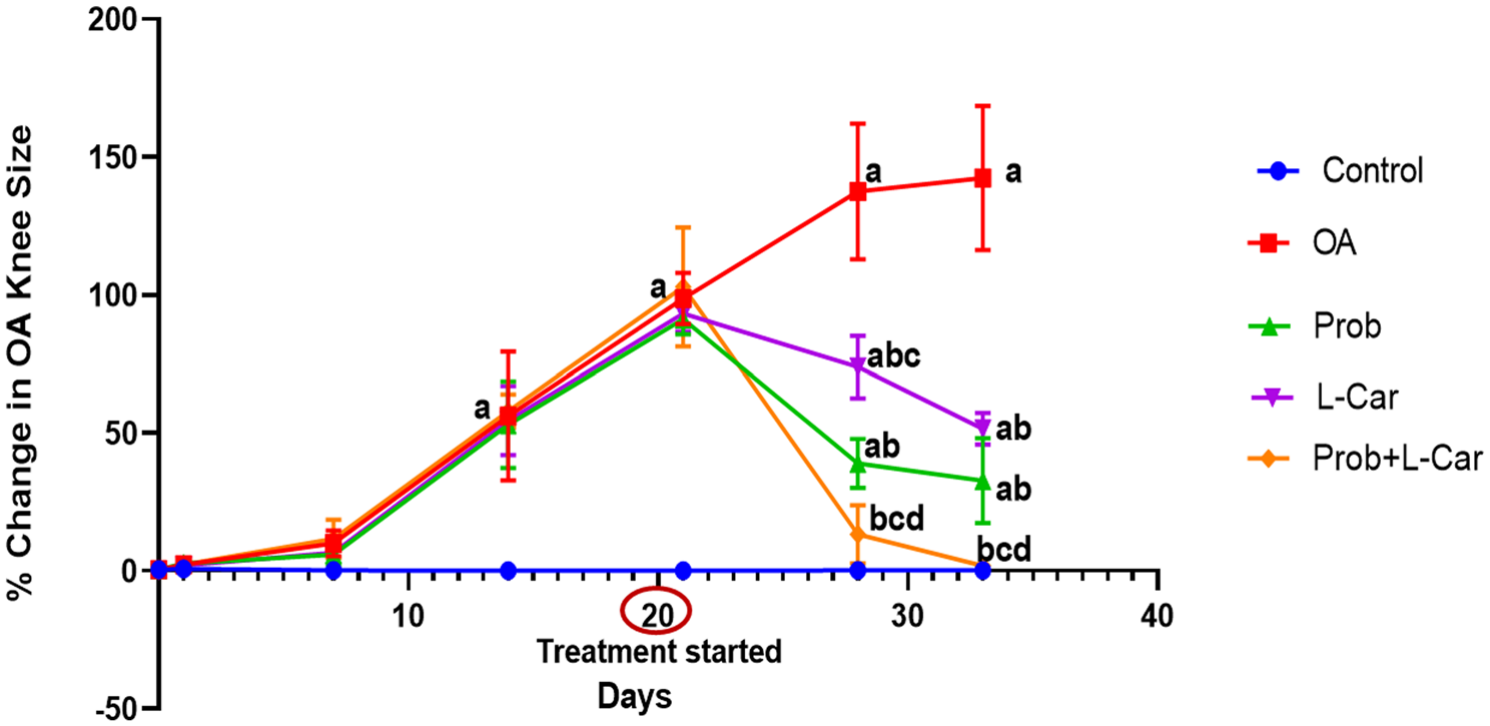
The % change in the OA (right) knee edema size from the normal knee (left) size on days 0,1,7,14,28,33 following treatment with probenecid at a dose of 50 mg/kg/day, l-carnitine at a dose of 100 mg/kg/day, and their combination for 14 days in all experimental groups after MIA induction of osteoarthritis in rats. Values are means ± S.D. of 6 rats. P < 0.05 vs. control rats (**a**), OA rats (**b**), Probenecid-treated rats (**c**), and L-Carnitine-treated rats (**d**)

The % change in OA (right) knee size from the normal knee (left) size on days 0,1,7,14, 21,28, and 33 is illustrated in Fig. 2. The intra-articular injection of MIA in the OA knee of all osteoarthritic untreated rats caused a significant continuous increase in the knee size revealing induction of inflammation and development of edema. All treatments given in this study caused a significant decline in the OA knee size on days 28 and 33. On day 33, there were no significant differences between the effects of probenecid- and l-carnitine-treated groups. The most significant reduction in the OA knee size was observed in the combination therapy that was not significantly different from the normal control rats at days 28 and 33.

### Probenecid and/or l-carnitine reversed the MIA-induced aberration in motor coordination and joint mobility

**Fig. 3:**
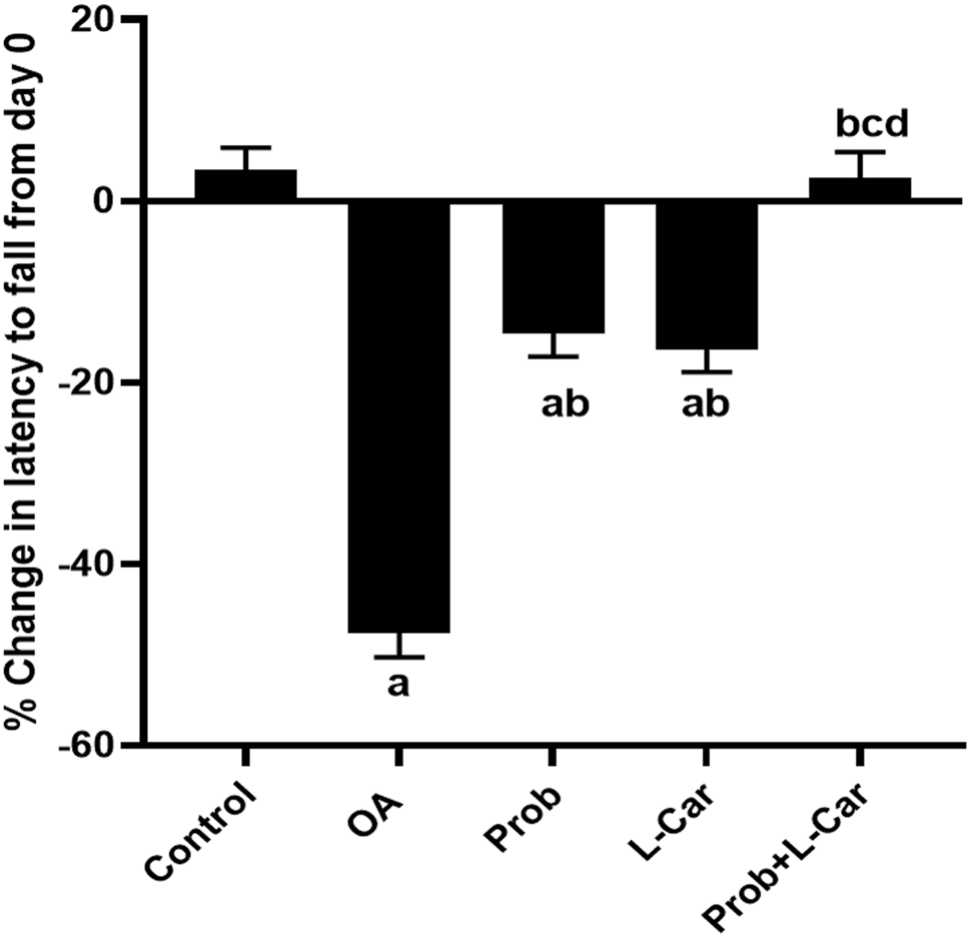
The % change in latency to fall on day 33 as compared to day 0 following treatment with probenecid at a dose of 50 mg/kg/day, l-carnitine at a dose of 100 mg/kg/day, and their combination for 14 days in all experimental groups after MIA induction of osteoarthritis in rats. Values are means ± S.D. of 6 rats. *P* < 0.05 vs. control rats (**a**), OA rats (**b**), Probenecid-treated rats (**c**), and L-Carnitine-treated rats (**d**)

Figure 3 depicts the percentage change in latency to fall on day 33 with reference to day 0. On day 33, the rotarod performance of the OA untreated rats was 47.6% ± 2.7 lower than on day 0. Furthermore, the drop-in performances were 14.6% ± 2.5 and 16.4% ± 2.5 in the probenecid and l-carnitine-treated groups, respectively, when compared to day 0. There were no significant differences between the probenecid and l-carnitine groups. Furthermore, as compared to day 0, the combination treatment group exhibited a 2.5% ± 2.9 improvement in performance, which was not substantially different from the normal control group’s performance rise 3.5% ± 2.3.

### Probenecid and/or l-carnitine attenuated the MIA-provoked P2X7R/NLRP3/cleaved caspase-1/IL-1β/IL-18 cue

**Fig. 4:**
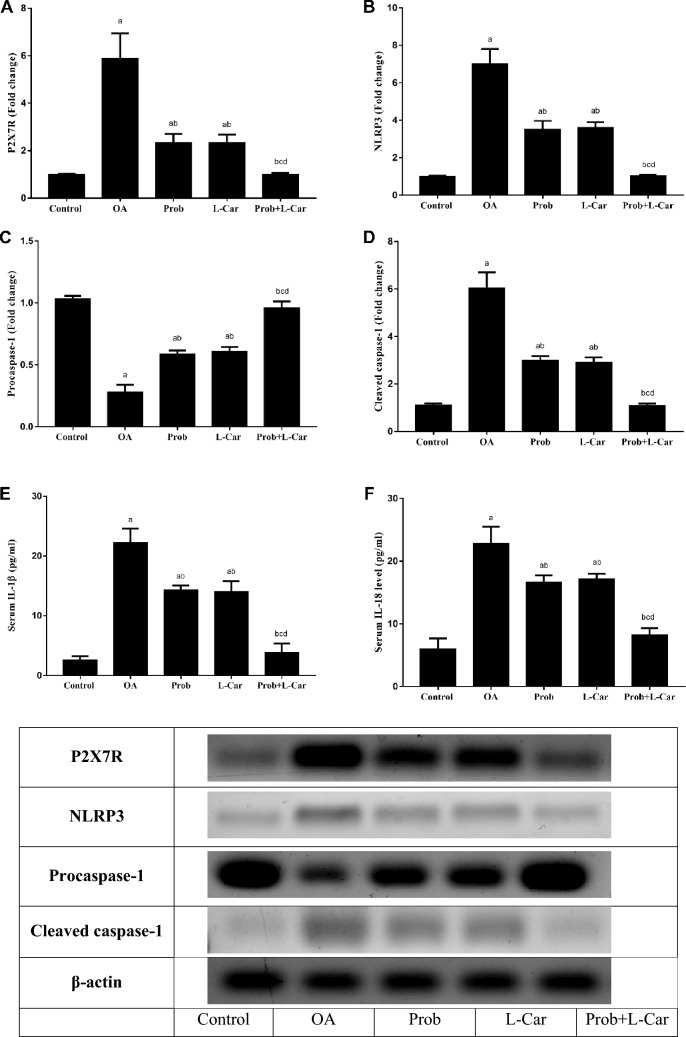
Change in knee cartilage expression of P2X7R (**A**), NLRP3 (**B**), procaspase-1 (**C**), cleaved caspase-1 (**D**) and serum levels of IL-1β (E), and IL-18 (**F**) following treatment with probenecid at a dose of 50 mg/kg/day, l-carnitine at a dose of 100 mg/kg/day, and their combination for 14 days in all experimental groups after MIA induction of osteoarthritis in rats. Values are means ± S.D. of 6 rats. P < 0.05 vs. control rats (**a**), OA rats (**b**), probenecid-treated rats (**c**), and L-Carnitine-treated rats (d). Western blot images shown are representative of one rat per group

As illustrated in Fig. 4, the OA rats showed increased protein expression of P2X7R (5.88 folds), NLRP3 (7.11 folds), and cleaved caspase-1 (5.48 folds) with a decreased expression of procaspase-1 (3.71 folds) in the knee joint cartilage as compared to control rats. In addition, OA induction significantly increased serum IL-1β (8.46 folds), and IL-18 (3.83 folds) as compared to the control group. Probenecid, L-carnitine treatments significantly declined protein expression of P2X7R (2.51, 2.52 folds), NLRP3 (2.00, 1.95 folds), and cleaved caspase-1 (2.02, 2.08 folds) as well as elevated the expression of procaspase-1 (2.11, 2.18 folds) in the knee joint cartilage as compared to OA rats, respectively. Also, serum IL-1β and IL-18 were significantly mitigated by probenecid treatment (1.57, 1.35 folds) as compared to the OA group, respectively. L-carnitine treatment possessed comparable results to that obtained by probenecid.

Meanwhile, the combination-treated group showed the best results in the aforementioned parameters, as there was a significant reduction in the protein expression of P2X7R (5.9 folds), NLRP3 (6.76 folds), and cleaved caspase-1 (5.58 folds) with augmented expression of procaspase-1 (3.45 folds) in the knee joint cartilage. The combination therapy significantly reduced serum IL-1β and IL-18 (5.79, 2.80 folds), as compared to OA rats, respectively.

### Probenecid and/or l-carnitine counteracted the MIA-augmented NF-κB activation and its downstream inflammatory cytokines

**Fig. 5:**
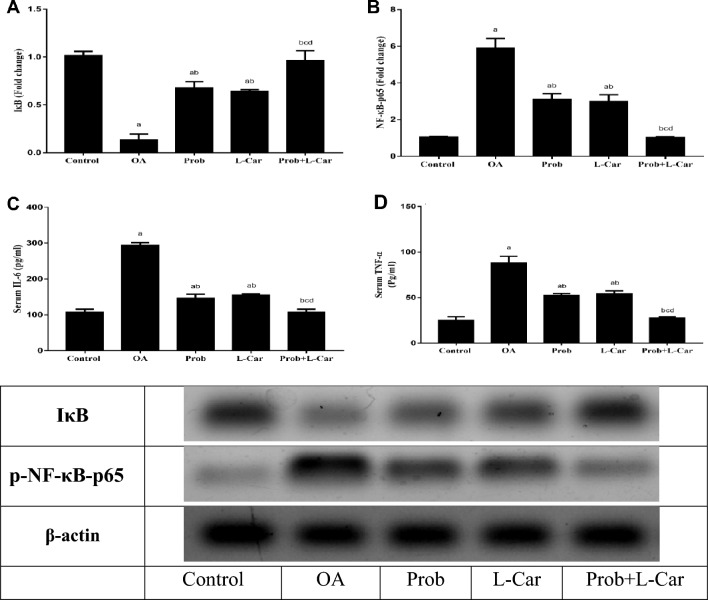
Change in knee cartilage expression of IκB (**A**), and NF-κB (**B**) and serum levels of IL-6 (**C**), and TNF-α (**D**) following treatment with probenecid at a dose of 50 mg/kg/day, l-carnitine at a dose of 100 mg/kg/day, and their combination for 14 days in all experimental groups after MIA induction of osteoarthritis in rats. Values are means ± S.D. of 6 rats. *P* < 0.05 vs. control rats (**a**), OA rats (**b**), Probenecid-treated rats (**c**), and L-Carnitine-treated rats (**d**). Western blot images shown are representative of one rat per group

As shown in Fig. 5, OA induction significantly decreased the expression of IκB (7.49 folds) as well as increased the expression of NF-κB (5.67 folds) in the knee joint cartilage of OA rats as compared to the control rats. Moreover, the OA model significantly increased serum IL-6 (2.74 folds) and TNF-α (3.49 folds) as compared to normal rats.

Treatment with probenecid or l-carnitine significantly elevated the IκB expression (4.99, 4.71 folds) as well as declined the expression of NF-κB (1.89, 1.98 folds) in the knee joint cartilage of treated rats as compared to OA rats. The combination therapy of both drugs caused the most significant elevation of IκB expression (7.08 folds) and reduction of NF-κB expression (5.75 folds) as compared to OA rats.

However, serum IL-6 and TNF-α levels were significantly ameliorated by probenecid and l-carnitine when compared to the untreated OA group to a comparable level respectively, but still significant when compared to the normal one, whereas the best results were witnessed in the combination-treated group to reach normal levels. The serum IL-6 and TNF-α levels were significantly declined by probenecid treatment (2.00, 1.67 folds), while l-carnitine declined their levels by (1.89, 1.61 folds), respectively. The combination therapy showed the most significant mitigation in the serum levels of IL-6 and TNF-α levels (2.74, 3.15 folds) as compared to OA rats.

### Probenecid and/or l-carnitine augmented MIA-attenuated miR-373 expression

**Fig. 6:**
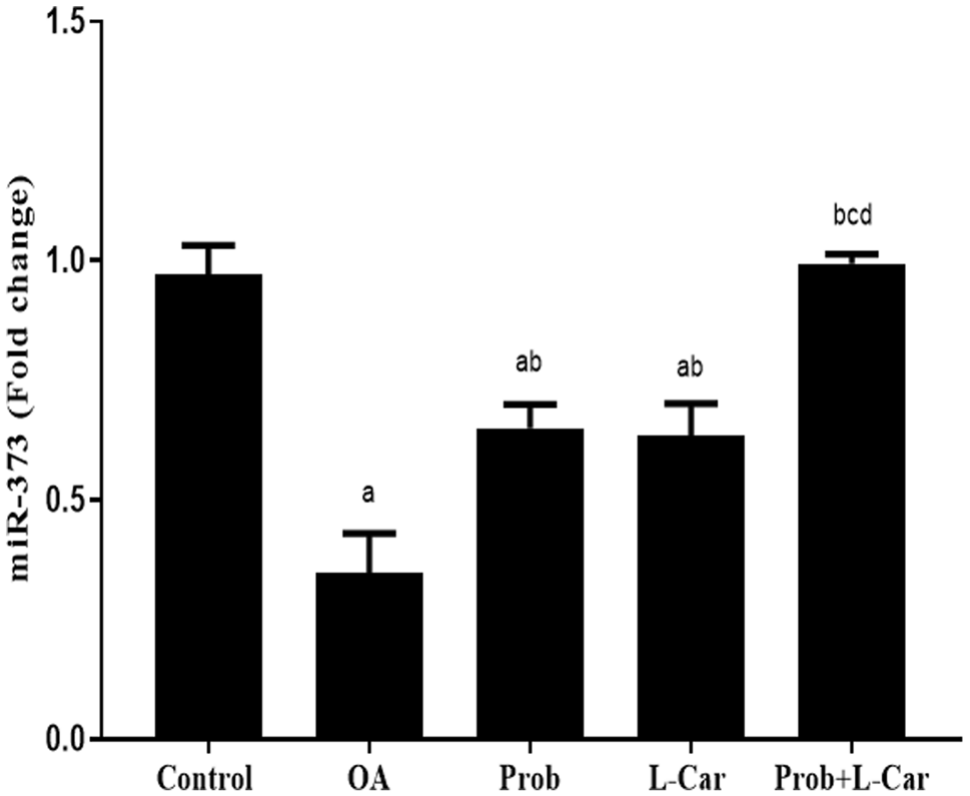
Change in knee cartilage miRNA-373 level following treatment with probenecid at a dose of 50 mg/kg/day, l-carnitine at a dose of 100 mg/kg/day, and their combination for 14 days in all experimental groups after MIA induction of osteoarthritis in rats. Values are means ± S.D. of 6 rats. *P* < 0.05 vs. control rats (**a**), OA rats (**b**), Probenecid-treated rats (**c**), and L-Carnitine-treated rats (**d**)

MiR-373 expression was significantly decreased in the OA rats (0.35 ± 0.08 fold change) as compared to control rats (0.97 ± 0.06 fold change), Fig. 6. Upon different treatments, its expression was significantly increased, whereas the effect of probenecid (0.65 ± 0.05 fold change) was comparable to that of l-carnitine (0.63 ± 0.07 fold change). The best effect was observed in the combination group (0.99 ± 0.02 fold change) to reach normal value; an effect that was more significant than either treatment alone.

### Probenecid and/or l-carnitine reversed the MIA-induced knee joints histopathological changes observed in H&E stained sections

**Fig. 7:**
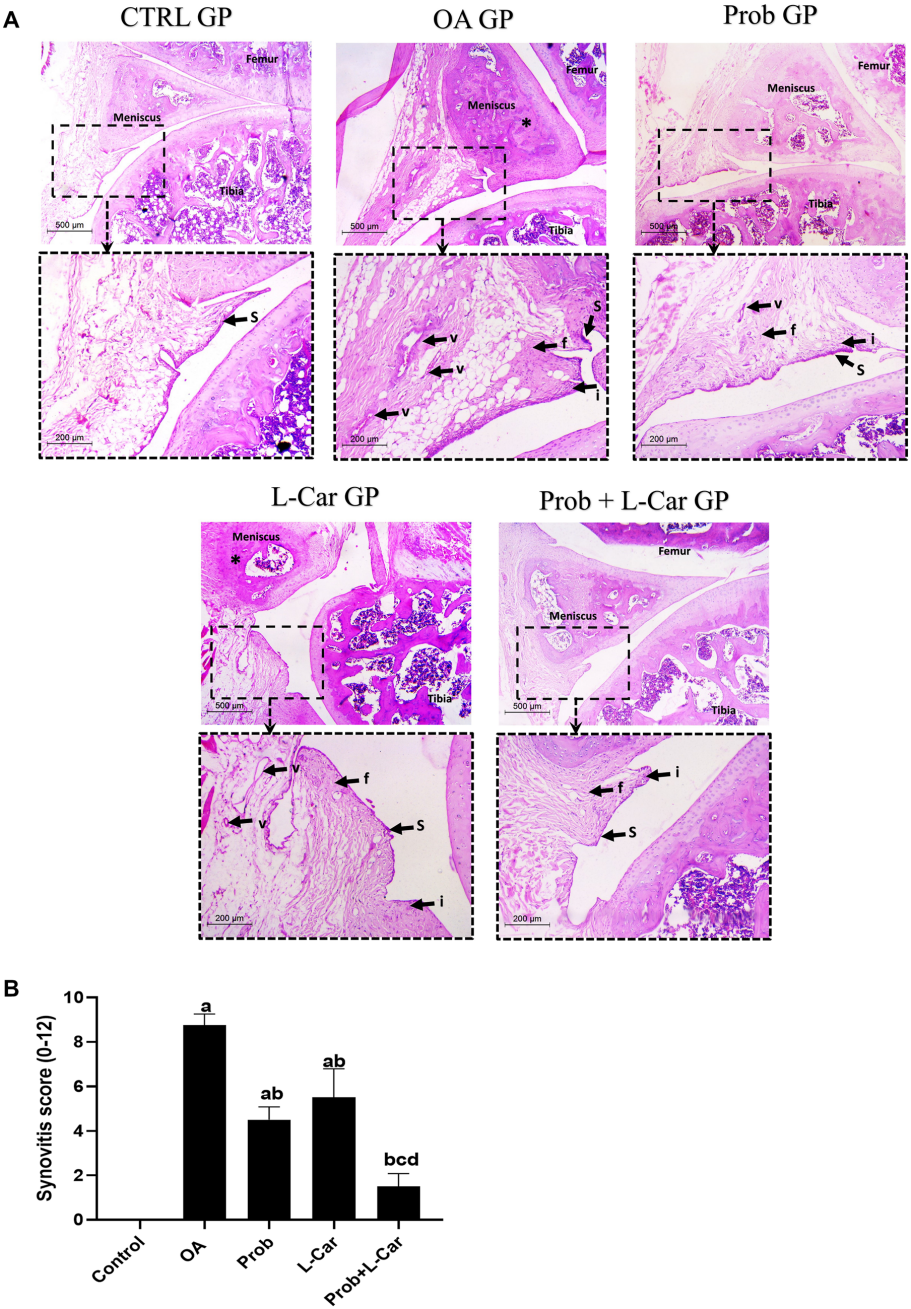
**A** Synovial membrane assessment in knee joint H&E stained sections following treatment with probenecid at a dose of 50 mg/kg/day, l-carnitine at a dose of 100 mg/kg/day, and their combination for 14 days in all experimental groups after MIA induction of osteoarthritis in rats, (× 40, scale bar = 500 microns, high power of squared area: × 100, scale bar = 200 microns). *S*  synovium, *i*  inflammation, *v*  vascularization, *f*  fibrosis, *meniscal sclerosis. CTRL GP shows thin synovium (s) with absent inflammation, vascularization, or fibrosis in sub-synovial tissue. OA GP shows synovial hyperplasia (s) with multiple foci of lymphoplasmacytic infiltrate (i) as well as fibrosis (**f**). also noted multiple dilated vascular spaces (v) indicating a high degree of synovitis. Osteosclerosis of the meniscus is seen (*) in the form of disfigurement of the meniscus and ossification. Prob GP shows improvement of three parameters with only mild synovial hyperplasia (s), inflammation (i) in addition to focal vascularization (v). L Car GP shows less improvement with residual meniscal sclerosis (*). Meanwhile, Prob + L CAR GP showed the best effect. Synovium is thin (s) and fibrosis is minimal (**f**). Occasional inflammation (i) is seen, and no vascular spaces are detected. Histology images shown are representative of one rat per group. **B** Total score of synovial membrane assessment in knee joint H&E stained Sects. (0–12) following treatment with probenecid at a dose of 50 mg/kg/day, l-carnitine at a dose of 100 mg/kg/day, and their combination for 14 days in all experimental groups after MIA induction of osteoarthritis in rats. The synovial membrane scoring method, which included (synovial membrane hyperplasia, lymphocytic/plasmacytic inflammation, sub-synovial fibrosis, and vascularity), was examined and scored (0–3) individually, yielding a total score of 12 (6 rats/group). *P* < 0.05 vs. control rats (**a**), OA rats (**b**), probenecid-treated rats (**c**), and l-carnitine-treated rats (**d**)

The histopathological assessment done for H&E stained sections of knee joints of different experimental groups is illustrated in Fig. 7A. The synovial membrane scoring method, which included (synovial membrane hyperplasia, lymphocytic/plasmacytic inflammation, sub-synovial fibrosis, and vascularity), was examined and scored (0–3) individually, yielding a total score of 12 as shown in Fig. 7B.

The control rats showed a preserved anatomical structure of the knee joint. Both femoral and tibial condyles were covered by smooth cartilage. The meniscus was regular in shape. The synovium was covered by one to two layers of synovial cells. Sub-synovial fat was free of inflammation, fibrosis, or vascular proliferation.

In contrast to the osteoarthritic untreated group, knee joints showed evident OA. The joint space was narrowed with irregular surfaces of both tibia and femur condyles. The meniscus showed disfigurement and sclerosis. Synovial hyperplasia was seen in addition to sub-synovial tissue fibrosis. Collections of mixed lymphoplasmacytic infiltrate and Large congested vascular spaces were noted.

Those changes were improved in treated groups. Probenecid-treated rats regained the anatomical integrity of knee joints. Mild synovial hyperplasia was seen with less fibrosis. Vascular spaces were only focally seen. L-carnitine group which showed residual meniscal sclerosis with mild fibrosis and congestion.

The combination of both therapies showed notable improvement. The smooth surface of both the tibia and femur as well as the meniscus was seen. The synovium was thin and formed of 2 layers only. Sub-synovial tissues showed only light occasional fibrosis with minimal inflammation. No vascular spaces were detected.

### Probenecid and/or l-carnitine reversed the MIA-induced knee joints histopathological changes observed in Safranin O-Fast green stained section

**Fig. 8:**
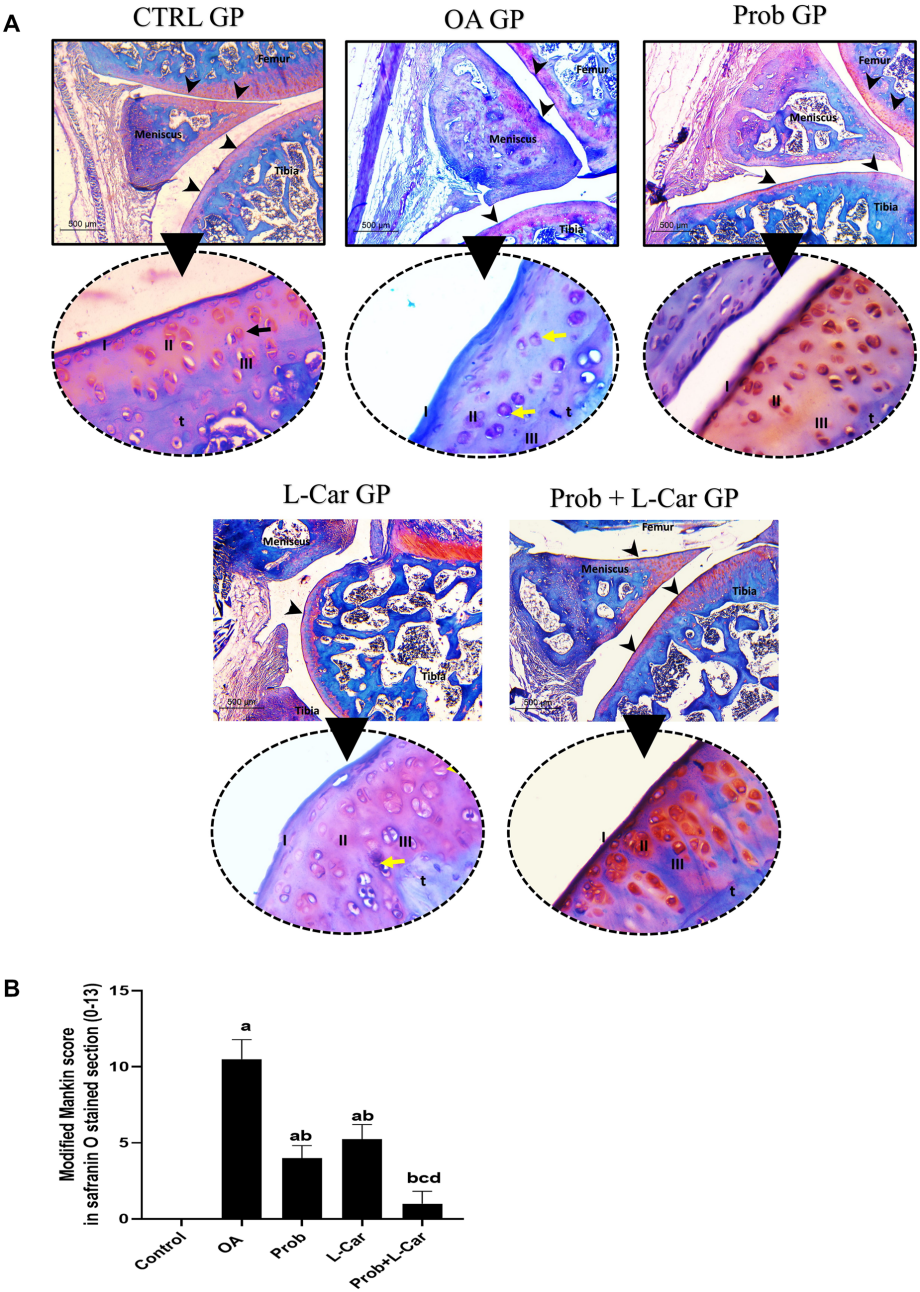
**A** Articular cartilage assessment in knee joint Safranin O-fast green stained sections following treatment with probenecid at a dose of 50 mg/kg/day, l-carnitine at a dose of 100 mg/kg/day, and their combination for 14 days in all experimental groups after MIA induction of osteoarthritis in rats, (× 40, scale bar = 500 microns, high power: × 400). Control GP shows a smooth surface of all bones (arrowheads) with deep red color indicating high proteoglycan content. High power view shows normal organization of chondrocytes into three superficial (I), mid (II) and deep zones (III) with intact tidemark (t). Black arrow is pointing at one viable chondrocyte. **OA GP** shows irregularities in articular surfaces (arrowheads) with sever proteoglycan depletion. High power shows evident hypocellularity and clustering of chondrocytes. Degenerated chondrocytes are noted (yellow arrow) and irregular tidemark (t). **Prob GP** shows a smooth articular surface and increased proteoglycan in the matrix (arrowheads). High power shows hypercellularity of cartilage with viable chondrocytes. L Car GP shows less improvement with residual focal surface irregularities (arrowhead). High power shows slightly irregular tidemark (t) and focal degenerated chondrocytes (yellow arrow). Meanwhile, Prob + L CAR GP showed the best effect. Articular cartilage is smooth (arrow head) with high proteoglycan content. In high power normal architecture and intact tide marks (t) are seen. Histology images shown are representative of one rat per group. arrowhead = articular surface, black arrow = viable chondrocyte, yellow arrow = degenerated chondrocyte, t = tidemark. I,II,III: represent different layers of articular cartilage. **B** Total Modified Mankin score in knee joint safranin O stained Sects. (0–13) following treatment with probenecid at a dose of 50 mg/kg/day, l-carnitine at a dose of 100 mg/kg/day, and their combination for 14 days in all experimental groups after MIA induction of osteoarthritis in rats. The scoring system includes 4 different parameters (cartilage structure, chondrocytes architecture, matrix staining, and tidemark integrity) with a total score out of 13 (6 rats/group). *P* < 0.05 vs. control rats (**a**), OA rats (**b**), probenecid-treated rats (**c**), and l-carnitine-treated rats (**d**)

Results of assessments of Safranin O-Fast green-stained sections are shown in Fig. 8A. Total Modified Mankin scores given for the safranin O stained sections of different experimental groups (0–13) are illustrated in Fig. 8B. Control group showed thick articular cartilage with smooth surface. On high-power examination, the cartilage layers were organized. It showed a superficial thin layer composed of flat cells and then a mid and deep zones of chondrocytes are present. All were embedded in a cartilaginous matrix showing a deep red colour indicating high proteoglycan content. The chondrocytes were viable. They were located within lacunae and showed large vesicular nuclei.

The OA untreated group showed thinned-out articular cartilage. The surface showed irregularities and abrasions. On high-power examination, the chondrocytes exhibited evident disorganization with areas of hypocellularity and clustering. The background showed marked proteoglycan depletion. Degenerated chondrocytes were seen in the form of dark-stained irregular nuclei or empty lacunae. Tidemark destruction was seen. The modified Mankin score was (10.5) indicating severe cartilage injury.

In the probenecid-treated group, the intra-peritoneal injection of probenecid had modified Mankin score (Chow and Chin 2020). The cartilage surface showed only mild irregularities. The chondrocytes partially regained their architecture however focal degenerated nuclei and hypercellularity were detected in some areas. Increased proteoglycan content was noted with intact tidemark. L-carnitine group showed also slight improvement in knee joint histopathology. The surface was smooth in some areas and slightly irregular in others. However, some disorganization of architecture and degenerated chondrocytes were still noted. Proteoglycan content showed a mild increase reaching a Mankin score of about [5.3].

Meanwhile, the combination of both treatments showed notable improvement in histopathology. The articular cartilage was smooth. The chondrocytes were of normal architecture with no clustering or degeneration. The matrix showed increased proteoglycan content. Tidemark integrity was retained. Those changes were evident in the modified Mankin score of this group (Yao et al. 2023).

### Statistical correlations

The Pearson coefficient test was used to analyze the link between miRNA-373 and P2X7R in all experimental groups, as well as correlations between P2X7R, NLRP3, and NF-B p65 in the Probenecid/L-carnitine treatment group. In all experimental groups, the statistical correlations demonstrated a negative association between miRNA-373 and P2X7R. In the Probenecid/L-carnitine-treated group, there was a positive correlation between P2X7R and NLRP3, as well as NF-B p65 Table 3.

**Table 3:** **A** Statistical correlations between miRNA-373 and P2X7R in all experimental groups; **B** Statistical correlations between P2X7R, NLRP3, and NF-κB p65 in the Probenecid/L-carnitine-treated group

A	miR-373			
Experimental Groups	OA GP	Prob GP	L-Car GP	Prob + L-Car GP
P2X7R	rs = – 0.67, p(2-tailed) = 0.22	rs = – 0.25, p(2-tailed) = 0.63	rs = – 0.22, p(2-tailed) = 0.72	rs = – 0.17, p(2-tailed) = 0.78

B	P2X7R			
Experimental Group	Prob + L-Car GP			
NLRP3	rs = 0.52, p(2-tailed) = 0.37			
NF-κB p65	rs = 0.98, p(2-tailed) = 0.003			

## Discussion

The existing OA pharmaceutical therapies have no discernible disease-modifying impact. In order to discover innovative, disease-modifying OA drugs to enhance patients’ quality of life, a new age of research must be launched exploring new targets (Vrouwe et al. 2021). As a result, the current study assessed the anti-inflammatory effects of probenecid and/or l-carnitine in attenuating OA via modulating the miR-373/P2X7/NLRP3/NF-kB cue.

Intraarticular injection of MIA in rats generated an OA rat model that matched human degenerative histopathological changes of OA. It was mentioned previously that MIA could inhibit glyceraldehyde-3-phosphate dehydrogenase (GAPDH) which is a key enzyme in glycolysis, leading to chondrocytes death (Takahashi et al. 2018).

The % change in OA (right) knee size from the normal (left) knee size on days 0, 1, 7, 14, 21, 28, and 33 was calculated. In OA untreated rats a continuous rise in knee size, showing inflammation and edema induced by MIA. By the end of the study on day 33, there were no significant differences in the effects of probenecid and l-carnitine treatment groups. The combination treatment resulted in the greatest reduction in OA knee size, which was not substantially different from the normal control rats. Prior investigations validated the potential of MIA to produce knee swelling as well as the ability of l-carnitine to attenuate knee swelling in MIA-induced knee OA in rats (Ali et al. 2016; Khodir et al. 2020).

An accelerated rotarod performance test was used to calculate the % change in performance on day 33 with reference to day 0. According to our findings, the performance of OA-untreated rats was significantly lower than that of normal rats. The probenecid or l-carnitine treatments were capable of decreasing the decline in performance, to almost the same extent. Treatment with both medications resulted in performance that was virtually equivalent to that of normal rats. Previously this test was done in the same osteoarthritic model as a measure of motor coordination and l-carnitine treatment was capable of significantly improving the rotarod performance as compared to OA rats (Khodir et al. 2020). Also, this test was used to assess motor balance in the traumatic OA model (Tsai et al. 2017).

The findings of our investigation showed that the level of miR-373 in the knee cartilage of osteoarthritic rats was significantly lower than that in normal rats. While probenecid or l-carnitine therapy significantly increased miR-373 level roughly equally. The combined treatment of both medications resulted in the greatest increase in miR-373 level. Our findings were consistent with prior research that found miR-373 was downregulated in the plasma and chondrocytes of OA patients (Zhang et al. 2018). Another investigation showed that the level of miR-373 was considerably reduced in OA chondrocytes compared to normal chondrocytes (Song et al. 2015).

The results of our study illustrated that there was a significant increase in the P2X7R expression in the cartilage of osteoarthritic rats as compared to normal rats. Statistically, our results showed a negative correlation between miR-373 and P2X7R. The inverse relationship between miR-373 and P2X7R was proved previously in osteoarthritic patients. Also, they illustrated that miR-373 could suppress OA by targeting P2X7R (8). Another previous study demonstrated that P2X7 receptor gene expression was provoked in OA as compared to healthy human knee cartilage. Moreover, it illustrated the capability of MIA to provoke the extracellular ATP, triggering the activation of P2X7R promoting inflammation and extracellular matrix degradation (29).

Additionally, our results showed that there was a significant elevation in the expression of NF-κB p65 and a decrease in the IκB in OA knee cartilages with a subsequent increase in the serum levels of IL-6 and TNF-α in OA rats as compared to normal rats. Moreover, the cartilage of osteoarthritic rats had provoked expression of NLRP3, cleaved caspase-1, and attenuated expression of procaspase-1 with subsequent increase in the serum IL-1β and IL-18. Furthermore, there was a positive statistical correlation between P2X7, NLRP3, and NF-κB p65 forming and endless loop of inflammation. This highlights the role of NF-κB and NLRP3 in pathogenesis in OA. Our results were consistent with previous study of the same rat model (Li et al. 2021).

Previously, it was stated that the P2X7 gates open in response to ATP binding, allowing Na^+^ and Ca^2+^ inflow together with K^+^ outflow, resulting in the activation of NLRP3 and, NF-κB p65. The NF-κB activation leads to an increase in the transcription and production of inflammatory cytokines that could furtherly activate of NF-κB creating a positive feedback loop (Hu et al. 2016; Li et al. 2021).

Furthermore, the activation of NF-κB could increase the transcription and production of NLRP3, pro-IL-1β, and Pro-IL-18. This may be considered as the first step in NLRP3 activation. The second step to finalize the activation of NLRP3 is an assembly of NLRP3 with procaspase 1 and ASC which is triggered by the activation of P2X7 receptors. Finally activated NLRP3 provoked the formation of cleaved caspase-1 to activate pro-IL-1β and pro-IL-18 to bioactive IL-1β and IL-18, leading to inflammation (Latz et al. 2013; Sharma and Kanneganti 2016; Franceschini et al. 2015).

Based on our findings and the findings of others, we concluded that MIA can increase ATP together with decreasing the amount of miR-373 to activate P2X7R. The P2X7R activates NF-κBP65 and NLRP3, which is coupled with the crosstalk between NF-κBP65 and NLRP3 to form an inflammatory loop. All of these factors act together to orchestrate the pathophysiology of OA.

Probenecid treatment significantly mitigated the P2X7R expression level nearly the same as l-carnitine. The most significant inhibition was observed in the combination therapy. An earlier study showed that probenecid could target the P2X7 receptor, to reduce hyperinflammation and severe influenza sickness in mice (Rosli et al. 2019). It could also affect the ATP release channel pannexin-1 (Silverman et al. 2008), which can directly interact with human and rodent P2X7, according to several research studies (Silverman et al. 2009; Pelegrin and Surprenant 2006). Probenecid, a clinically utilized broad-spectrum Panx1 blocker, significantly reduced mechanical allodynia in a model of posttraumatic OA (Mousseau et al. 2018).

Moreover, treatment with probenecid significantly attenuated the expression of NF-κB p65, NLRP3, cleaved caspase-1, and significantly provoked the expression of IκB and procaspase-1. Also, the serum cytokine levels including IL-6, IL-18, IL-1β, and TNF-α were significantly attenuated along with probenecid treatment. There was no significant difference between the effect observed with probenecid or l-carnitine treatment. The group receiving the combination treatment had the most notable impact.

It had been previously mentioned that probenecid therapy, protects against early brain injury after subarachnoid hemorrhage in rats via causing a marked reduction in the expression of P2X7R due to the inhibition of the pannexin-1 channel. Additionally, probenecid was able to reduce the production of inflammatory cytokines, prevent the activation of the AIM2 inflammasome, and enhance neurological functioning (Zheng et al. 2022). In another study, probenecid protected the primary astrocytes from oxygen–glucose deprivation damage via modulating inflammasome activity (Jian et al. 2016).

In a prior study, l-carnitine inhibited the activation of NF-κB, which improves the oxidative stress response to angiotensin II and protected against the invitro liver damage model (Blanca et al. 2017; Nakamura et al. 2018). Furthermore, it was shown to be effective at reducing the synovial fluid IL-1β in rats with monoiodoacetate-induced knee OA (Ali et al. 2016). Moreover, acetyl l-carnitine ameliorated the depressive-like anxiety behaviors induced by LPS by causing a decline in the NF-κB and NLRP3 inflammasome expression (Samin et al. 2021).

In our work, histopathological examination of a knee joint segment stained with H and E revealed that the anatomical structure of the knee joint was intact in the control rats. While the osteoarthritic untreated group revealed obvious OA. The uneven surface of the tibia and femur condyles restricted the joint space. The meniscus was disfigured and sclerosis was present. In addition to sub-synovial tissue fibrosis, synovial hyperplasia was seen. There were collections of mixed lymphoplasmacytic infiltration and large congested vascular areas (Hamdalla et al. 2022; Johnson et al. 2022). Also, the total score given upon assessment of the synovial membrane of knee joint H and E stained sections was significantly increased in OA rats as compared to normal rats, indicating the occurrence of hyperplasia and inflammation. The treatment of both probenecid and l-carnitine significantly reduced the aforementioned score nearly toward normal state.

Safranin O-Fast green stained sections showed that the normal thick smooth articular cartilage with a normal level of chondrocytes and proteoglycan in the control group. The untreated OA group had thinned articular cartilage with surface abrasions. This was coupled with the observation of disorganized chondrocytes and depletion of proteoglycan (Lu et al. 2018; Salman et al. 2019). The total modified Mankin score given upon assessment of the articular cartilage of knee joint safranin-O-fast green stained sections was significantly increased in OA rats as compared to normal rats, indicating the occurrence of cartilage erosion and decrease in proteoglycan content (Jeong et al. 2017).

All of the therapies improved the anomalies identified in the H and E as well as Safranin O-Fast green OA knees stained sections, with the combination of both medicines having the most substantial impact. In addition to lowering the overall score of the synovial membrane in H/E-stained sections and the total modified Mankin score of articular cartilage in safranin O fast green-stained sections.

## Conclusion

L-carnitine augmented the probenecid’s anti-inflammatory effect to attenuate MIA-induced OA in rats by provoking the miRNA-373 level and inhibiting the P2X7/NLRP3/NF-κB milieu. This led to a subsequent decrease in the serum levels of inflammatory cytokines (IL-1β, IL-18, IL-6, TNF-α). Also, the combination therapy had the most prominent effect on reducing the OA knee size, enhancing the motor balance, and reversing the abnormalities observed in the H and E as well as Safranin O-Fast green OA knees stained sections. The most significant reductions in the OA knee were observed in the combination therapy that was not significantly different from the normal control rats. But significantly different from either treatment alone. This illustrates the significance and importance of the novel combination in the treatment of OA.

## Data Availability

All data generated or analyzed during this study are included in this published article.
