# Correction: L-Carnitine augments probenecid anti-inflammatory effect in monoiodoacetate-induced knee osteoarthritis in rats: involvement of miRNA-373/P2X7/NLRP3/NF-κB milieu

**DOI:** 10.1007/s10787-023-01410-x

**Published:** 2023-12-30

**Authors:** Rawan Mahfouz, Safaa H. El-Rewini, Asser I. Ghoneim, Eman Sheta, Mennatallah A. Ali, Sherihan Salaheldin Abdelhamid Ibrahim

**Affiliations:** 1 Department of Pharmacology and Therapeutics, Faculty of Pharmacy, Pharos University in Alexandria (PUA), Canal El- Mahmoudia Street, Smouha, Alexandria Egypt; 2 Department of Pharmacology, Faculty of Medicine, Alexandria University, Alexandria, Egypt; 3 Department of Pharmacology and Toxicology, Faculty of Pharmacy, Damanhour University, Damanhour, Egypt; 4 Department of Pathology, Faculty of Medicine, Alexandria University, Alexandria, Egypt

**Correction: Inflammopharmacology** 10.1007/s10787-023-01376-w

It has come to our attention that there are 2 typing mistakes, one in the abstract and one in the findings. The typing mistakes are highlighted in the text.

The corrected abstract should read:

Osteoarthritis (OA) is a degenerative joint disease, whereas the underlying molecular trails involved in its pathogenesis are not fully elucidated. Hence, the current study aimed to investigate the role of miRNA-373/P2X7/NLRP3/NF-κB trajectory in its pathogenesis as well as the possible anti-inflammatory effects of probenecid and l-carnitine in ameliorating osteoarthritis via modulating this pathway. In the current study, male Sprague Dawley rats were used and monoiodoacetate (MIA)-induced knee osteoarthritis model was adopted. Probenecid and/or L-carnitine treatments for **14 days** succeeded in reducing OA knee size and reestablishing motor coordination and joint mobility assessed by rotarod testing. Moreover, different treatments suppressed the elevated serum levels of IL-1β, IL-18, IL-6, and TNF-α via tackling the miRNA-373/P2X7/NLRP3/NF-κB, witnessed as reductions in protein expressions of P2X7, NLRP3, cleaved caspase-1 and NF-κB. These were accompanied by increases in procaspase-1 and IκB protein expression and in miRNA-373 gene expression OA knee to various extents. In addition, different regimens reversed the abnormalities observed in the H and E as well as Safranin O-Fast green OA knees stained sections. Probenecid or l-carnitine solely showed comparable results on the aforementioned parameters, whereas the combination therapy had the most prominent effect on ameliorating the aforementioned parameters. In conclusion, l-carnitine augmented the probenecid’s anti-inflammatory effect to attenuate MIA-induced osteoarthritis in rats by provoking the miRNA-373 level and inhibiting the P2X7/NLRP3/NF-κB milieu, leading to the suppression of serum inflammatory cytokines: IL-1β, IL-18, IL-6, and TNF-α. These findings suggest the possibility of using probenecid and l-carnitine as a useful therapeutic option for treatment of osteoarthritis.

The corrected results (Probenecid and/or l-carnitine reversed the MIA-induced knee joints histopathological changes observed in Safranin O-Fast green stained section) should read:

In the probenecid-treated group, the **intra-peritoneal** injection of probenecid had modified Mankin score (Chow and Chin 2020). The cartilage surface showed only mild irregularities. The chondrocytes partially regained their architecture however focal degenerated nuclei and hypercellularity were detected in some areas. Increased proteoglycan content was noted with intact tidemark. L-carnitine group showed also slight improvement in knee joint histopathology. The surface was smooth in some areas and slightly irregular in others. However, some disorganization of architecture and degenerated chondrocytes were still noted. Proteoglycan content showed a mild increase reaching a Mankin score of about [5.3].

The original article has been corrected.

